# Novel Halotolerant Bacteria from Saline Environments: Isolation and Biomolecule Production

**DOI:** 10.3390/biotech14020049

**Published:** 2025-06-19

**Authors:** Simona Neagu, Mihaela Marilena Stancu

**Affiliations:** Institute of Biology Bucharest of Romanian Academy, 296 Splaiul Independentei, 060031 Bucharest, Romania

**Keywords:** saline environments, halotolerant bacteria, biomolecules, antimicrobial activity

## Abstract

Microorganisms from saline environments have garnered significant interest due to their unique adaptations, which enable them to thrive under high-salt conditions and synthesize valuable biomolecules. This study investigates the biosynthesis of biomolecules, such as extracellular hydrolytic enzymes, biosurfactants, and carotenoid pigments, by four newly halotolerant bacterial strains isolated from saline environments in the Băicoi (soil, water) and Curmătura (mud) area (Prahova County, Romania). Isolation was performed on two selective culture media with different NaCl concentrations (1.7 M, 3.4 M). Based on their phenotypic and molecular characteristics, the four halotolerant bacteria were identified as *Halomonas elongata* SB8, *Bacillus altitudinis* CN6, *Planococcus rifietoensis* CN8, and *Halomonas stenophila* IB5. The two bacterial strains from the *Halomonas* genus exhibited growth in MH medium containing elevated NaCl concentrations (0–5 M), in contrast to the other two strains from *Bacillus* (0–2 M) and *Planococcus* (0–3 M). The growth of these bacteria under different salinity conditions, hydrocarbon tolerance, and biomolecule production were assessed through biochemical assays, spectrophotometry, and high-performance thin-layer chromatography. The antimicrobial properties of biosurfactants and carotenoids produced by *H. elongata* SB8, *B. altitudinis* CN6, *P. rifietoensis* CN8, and *H. stenophila* IB5 were evaluated against four reference pathogenic microorganisms from the genera *Escherichia*, *Pseudomonas*, *Staphylococcus*, and *Candida*. *H. elongata* SB8 showed the highest hydrocarbon tolerance. *B. altitudinis* CN6 exhibited multiple hydrolase activities and, along with *H. elongata* SB8, demonstrated biosurfactant production. *P. rifietoensis* CN8 produced the highest carotenoid concentration with antifungal and antimicrobial activity. Exploring these organisms opens new pathways for bioremediation, industrial bioprocessing, and sustainable biomolecule production.

## 1. Introduction

The study of microorganisms inhabiting saline environments has gained significant attention in recent decades due to the incomplete characterization of these communities [1,2]. Microorganisms that survive in saline environments can be found in various natural ecosystems, such as salt lakes, saline soils, sabkhas, soda lakes, saline springs, and salt mines. They are also present in anthropogenic or industrial habitats such as brine ponds, salt ponds, salted foods, and oil fields. Studying these microorganisms provides valuable insights into the biological adaptations and survival mechanisms that enable life under such extreme conditions [3,4,5]. These environments were traditionally considered habitats with low bacterial diversity, predominantly colonized by archaea [1,2]. Microorganisms from saline environments can be classified based on their salt concentration requirements into several categories: slight halophiles (0.2–0.5 M or 1–3% NaCl), moderate halophiles (0.5–2.5 M or 3–15% NaCl), extreme halophiles (2.5–5.2 M or 15–30% NaCl), and halotolerant, which do not have an absolute requirement for salt but can tolerate high salt concentrations [3,6].

Adaptation to ionic and osmotic stress due to high salt concentrations is achieved through the compatible salt-in strategy and compatible solute strategy [7,8]. The salt-in strategy involves the accumulation of high concentrations of inorganic ions, particularly K^+^, in the cytoplasm. Na^+^ is largely excluded from the cytoplasm to prevent ionic toxicity and maintain osmotic balance between the external hypersaline environment and the cell interior. This results in an elevated concentration of K^+^ inside the cell, necessitating protein adaptation to ensure their proper function. These proteins have a higher proportion of acidic amino acids, conferring a negative charge and enhancing their solubility and stability in environments with high ionic strength. This adaptation mechanism is primarily observed in extremely halophilic archaea from the Halobacteriaceae family, although it is also present in certain extremely halophilic bacteria that have developed archaeal traits. For example, the extremely halophilic bacterium *Salinibacter ruber* utilizes a characteristic sulfonolipid, accumulates potassium inside the cell, and produces pigments, features that are typically associated with halophiles from the domain Archaea [3]. The compatible solute strategy, commonly observed in halotolerant bacteria, involves accumulating small organic molecules known as osmolytes. These solutes help maintain cellular osmotic balance without disrupting metabolic processes. The main types of compatible solutes identified in halotolerant and moderately halophilic microorganisms include polyols, sugars, amino acids, and their derivatives, as well as glycine, betaine, and ectoine [9,10].

Due to these adaptations, halophilic and halotolerant bacteria are attractive from a biotechnological point of view, having the ability to produce a wide range of valuable biomolecules, including extracellular hydrolytic enzymes, biosurfactants, exopolysaccharides, biopolymers, and carotenoid pigments, as well as compatible solutes [11,12]. Among these, extracellular enzymes are particularly valued for their capacity to remain active under extreme pH, temperature, and salinity conditions, which makes them suitable for industrial applications [13,14,15]. Biosurfactants produced by these bacteria facilitate the solubilization of hydrophobic compounds, making them highly valuable in the bioremediation of petroleum hydrocarbon-contaminated environments [16]. In turn, exopolysaccharides contribute to protection against dehydration and facilitate biofilm formation, with applications in the food and pharmaceutical industries [17,18]. Carotenoid pigments are secondary metabolites produced by a wide range of microorganisms, including halophilic and halotolerant species. In these bacteria, carotenoids contribute to adaptation under extreme environmental conditions, offering protection against oxidative stress, dehydration, and UV radiation [19]. They also play a structural role by stabilizing cell membranes under saline stress and contribute to the pigmentation of bacterial colonies [20,21]. Most carotenoids synthesized by bacteria belong to the C40 group, such as β-carotene, lycopene, and zeaxanthin, known for their antioxidant and photoprotective properties, with potential applications in the pharmaceutical, cosmetic, and food industries [22,23]. In comparison, some halophilic archaea synthesize C50 carotenoids like bacterioruberin, a membrane-associated pigment with antioxidant properties and a conjugated structure that contributes to cellular protection under hypersaline and high-radiation conditions [24,25]. While some haloarchaea can also produce extremozymes with industrial relevance, particularly proteases and nucleases, their capacity to generate biosurfactants remains largely underexplored.

With the increasing demand for natural carotenoids, research into identifying natural sources has intensified. One advantage of using microorganisms as producers of carotenoids is the ability to optimize production by controlled cultivation, which can decrease synthesis time and improve efficiency [26]. This study investigates the biosynthesis of biomolecules, such as extracellular hydrolytic enzymes, biosurfactants, and carotenoid pigments, by four newly halotolerant bacterial strains belonging to *Halomonas*, *Bacillus,* and *Planococcus* genera isolated from saline environments in the Băicoi (i.e., soil, water) and Curmătura (i.e., mud) area, Prahova County, Romania. After optimization of the culture conditions, morphological, biochemical, and molecular characterization of the halophiles was conducted. The bioactive properties of biosurfactant and carotenoid extracts were also evaluated, focusing on their antimicrobial activity against four reference pathogenic microorganisms from the genera *Escherichia*, *Pseudomonas*, *Staphylococcus*, and *Candida*.

## 2. Materials and Methods

### 2.1. Sample Collection and Physicochemical Characterization

The soil, mud, and water samples analyzed in this study were collected (in July 2024) from two different salt wells located in Băicoi town (Latitude: 45°2′34.553″ N, Longitude: 25°53′18.667″ E, Altitude: 265 m) and Curmătura village (Latitude: 45°09′25.307″ N, Longitude: 26°09′06.006″ E, Altitude: 213 m), both located in Prahova County, Romania, a region rich in salt deposits, petroleum, and natural gas resources. Soil samples were obtained from three distinct points near the brine wells, each taken from a depth of 10 cm, while the mud sample was collected from the surface layer, where a visible salt crust was observed. After collection, the samples were immediately brought to the laboratory for further processing. The physical and chemical parameters of the samples, including temperature, pH, density, and salinity, were determined according to the method described by Enache et al. [27]. The chloride content was measured titrimetrically using the Mohr method [28].

### 2.2. Isolation, Characterization, and Identification of Halophiles

**Isolation.** Two growth media were used for the isolation and cultivation of halophilic microorganisms.

The MH medium [29] contained the following (g/L): NaCl, 100; KCl, 2; MgCl_2_∙6H_2_O, 7; MgSO_4_∙7H_2_O, 9.6; CaCl_2_∙2H_2_O, 0.36; NaHCO_3_, 0.06; NaBr, 0.026; glucose, 1; proteose-peptone, 5; and yeast extract, 10. The culture medium JCM 168 (Rasooli et al., 2016) [30] contained the following (g/L): NaCl, 200; KCl, 2; MgSO_4_∙7H_2_O, 20; FeCl_2_∙4H_2_O, 0.036; MnCl_2_∙4H_2_O, 0.00036; casamino acids, 5; yeast extract, 5; sodium glutamate, 1; and trisodium citrate, 3. MH agar and JCM 168 agar were prepared by supplementing their respective liquid media with 20 g of agar. The pH of the culture media was adjusted to 7.2. Decimal dilutions of the samples were prepared in sterile saline solutions containing 10% and 20% salt. Then, 1 mL of each decimal dilution (10^−1^, 10^−2^, and 10^−3^) was inoculated in triplicate by the pour-plate method [31] in both MH and JCM 168 medium. Plates containing MH medium for moderately halophilic bacteria and fungi were incubated at 30 °C for 10 days, while plates with JCM 168 medium for extremely halophilic bacteria and archaea isolation were incubated at 37 °C for 20 days. Throughout the study, all experiments were conducted in duplicate. The number of viable microorganisms in the samples was expressed as colony-forming units (CFU/mL or CFU/mg). The new strains were purified through repeated passages on the isolation media and preserved in glycerol at −80 °C.

**Phenotypic and molecular characteristics.** The isolates were identified based on their phenotypic characteristics (i.e., the color of the colony, temperature growth, salt tolerance capacity, Gram staining, cell shape, motility, respiratory type, catalase, oxidase, and extracellular hydrolytic enzyme production) [32] and molecular characteristics. Genomic DNA was extracted from cultures using the Pure Link genomic DNA kit (Invitrogen, Carlsbad, CA, USA).

Polymerase chain reaction (PCR) amplification of the 16S rRNA gene was performed using genomic DNA, universal primers 27f/1492r [33] or 20f/1492r [34], and GoTaq G2 hot start polymerase (Promega, Madison, WI, USA), following the protocol described by Stancu (2020, 2023) [35,36]. Thermal conditions were set using an Eppendorf Mastercycler Pro S thermocycler (Hamburg, Germany) and consisted of an initial cycle at 94 °C for 10 min and 35 cycles of 94 °C for 1 min, 55 °C for 30 s, and 72 °C for 2 min, with a final cycle at 72 °C for 10 min. The reaction products were analyzed on 1.5% (*w*/*v*) agarose gels stained with SYBR Safe (Invitrogen, Carlsbad, CA, USA) [37,38]. The PCR products of the 16S rRNA gene were gel-purified to isolate the specific 1465 bp band, which was subsequently subjected to sequencing at Macrogen Europe (Amsterdam, Netherlands). The 16S rRNA gene sequence was compared to those from related organisms available in the GenBank database using the BLAST program (https://blast.ncbi.nlm.nih.gov/Blast.cgi), accessed on 25 February 2025. The alignment was performed using the default parameters of BLASTn (version 2.16.0).

Random amplification of polymorphic DNA (RAPD) was performed using genomic DNA, primers AP12 or AP5, and GoTaq G2 hot start polymerase (Promega, Madison, WI, USA) [35]. Thermal conditions consisted of an initial cycle at 94 °C for 10 min, followed by 45 cycles of 94 °C for 1 min, 36 °C for 1 min, and 72 °C for 2 min, with a final cycle at 72 °C for 10 min. The reaction products were analyzed on 2% (*w*/*v*) agarose gels, stained with SYBR Safe (Invitrogen, Carlsbad, CA, USA) [37].

**Halophily and halotolerance.** The NaCl requirement for the growth of the isolates was assessed by spot-inoculating cultures (20 μL) onto MH agar plates supplemented with NaCl concentrations ranging from 0 M to 5 M. The Petri plates were incubated at 30 °C and 37 °C for 1–5 days. To evaluate growth kinetics, cultures were inoculated at a 1:9 (*v*/*v*) ratio into MH-NaCl broth containing the same NaCl concentrations. Growth was monitored spectrophotometrically at 660 nm for 24 h at 30 °C using a SpectraMax ABS Plus microplate reader (Molecular Devices, San Jose, CA, USA). Cell viability was determined using 0.3% (*w*/*v*) 2,3,5-triphenyl tetrazolium chloride (TTC). Uninoculated MH-NaCl broth was used as a control.

**Hydrocarbon tolerance.** The tolerance of the isolates to hydrocarbons (i.e., diesel, kerosene, *n*-hexane, and *n*-hexadecane) was evaluated using the hydrocarbon overlay agar method [35,36,37,38]. Cultures were spot-inoculated (20 μL) onto MH agar, air-dried, and overlaid with hydrocarbons for one hour, and then the hydrocarbons were removed. The Petri plates were incubated at 30 °C for 1–5 days. Hydrocarbon tolerance was evaluated by comparing colony formation to the control, which was prepared under identical conditions but without hydrocarbons. To assess growth kinetics, cultures were inoculated at a 1:9 (*v*/*v*) ratio into MH broth supplemented or not with 10% (*v*/*v*) hydrocarbons. Growth was monitored spectrophotometrically at 660 nm for 24 h at 30 °C using a SpectraMax ABS plus microplate reader (Molecular Devices, San Jose, CA, USA). Cell viability was determined using 0.3% (*w*/*v*) TTC [38]. Uninoculated MH broth was used as a control.

### 2.3. Biomolecule Production by Halophiles

**Extracellular hydrolytic enzymes.** The ability of isolates to synthesize extracellular hydrolases (i.e., lipase, protease, amylase, cellulase, xylanase, and pectinase) was assessed using the plate assay method [39]. MH and JCM 168 media compositions were modified to target specific enzymatic activities. In the MH medium, glucose and proteose peptone were omitted. In the JCM 168 medium, casamino acids were excluded, and the concentration of yeast extract was reduced from 5 to 1 g/L. Enzymatic substrates, including Tween-80, casein, starch, carboxymethylcellulose (CMC), xylan, and pectin, were added at 1 g/L. After spot inoculating the cultures onto modified MH and JCM 168 media, Petri plates were incubated at 30 °C and 37 °C for 10 to 20 days. The results were interpreted as follows: proteolytic, amylolytic, carboxymethylcellulolytic, xylanolytic, and pectinolytic activities were indicated by the formation of clear zones surrounding the cultures after treatment with a 0.3–0.6% I_2_–KI solution for amylase and pectinase or a 0.1% Congo red solution for cellulase and xylanase. Conversely, lipolytic activity was evidenced by the presence of an opaque halo around the cultures.

**Biosurfactants.** The halophiles’ biosurfactant production was evaluated using the emulsification index, the methylene blue method, a CTAB agar plate, and HPTLC analysis.

Emulsification index. The cell-free supernatant was combined with diesel in a 1:1 (*v*/*v*) ratio and vortexed (3 min). The emulsification index (*E*_24_) was established after a 24 h incubation [35,38].

Methylene blue method. The cell-free supernatant was mixed with 0.03% (*w*/*v*) methylene blue and chloroform in a 1:1 (*v*/*v*) ratio and then vortexed (3 min). After 30 min, the optical density of the organic phase was measured at 625 nm using a SPECORD 200 UV–Vis spectrophotometer (Analytik Jena, Jena, Germany) as described by Stancu [36,37,38].

Cetyltrimethylammonium bromide (CTAB) agar plate method. Cultures and cell-free supernatants (20 μL) were spotted on CTAB methylene blue agar [36,37,38] or filter paper discs, air-dried, and incubated at 30 °C for 1–5 days. Rhamnolipid-producing cultures formed a dark blue halo.

High-performance thin-layer chromatography (HPTLC). The cell-free supernatants were subjected to extraction of biosurfactants using a chloroform–methanol solvent mixture in a 2:1 (*v*/*v*) ratio. The extracts were concentrated using an Eppendorf vacuum concentrator (Hamburg, Germany) to evaporate the solvent. HPTLC was subsequently performed on these biosurfactant extracts using the CAMAG TLC system (Muttenz, Switzerland). Samples were applied under a nitrogen stream onto silica gel glass plates (Merck, Darmstadt, Germany) and developed using a chloroform–methanol–water mixture (65:25:4, *v*/*v*/*v*) as the mobile phase [37,38]. After separation, derivatization with iodine vapors was performed to visualize lipid components or samples were treated with a solution of 0.2% orcinol in 53% sulfuric acid to detect sugars within the biosurfactant molecules. The TLC plate was examined and scanned under ultraviolet light (366 nm) and visible light (500 nm).

**Carotenoid pigments.** For pigment extraction, the cell pellets were washed twice and then pigments were extracted in the dark with acetone. The extracts were concentrated using an Eppendorf vacuum concentrator (Hamburg, Germany) to evaporate the solvent and weighed. Carotenoid extracts were analyzed using UV–visible scanning spectra recorded from 300 to 800 nm with a SPECORD 200 UV–visible spectrophotometer (Analytik Jena, Jena, Germany). The total content of carotenoids in the acetone extract was assessed by measuring the absorbance of the sample at 494 nm using a cuvette with a 1 cm path length. The concentration was calculated using the specific absorption coefficient of 2500, as described by Hiyama et al. [40]. Carotenoid extracts were also analyzed using HPTLC with a CAMAG TLC system. Samples were applied under a nitrogen stream onto silica gel glass plates and developed with a chloroform–methanol mixture (90:10, *v*/*v*) as the mobile phase [35]. The TLC plate was examined and scanned under ultraviolet light (366 nm).

**Antimicrobial activity.** The antimicrobial activity of the biosurfactant and carotenoid extracts was analyzed by the spot-on-lawn method [41]. Four reference pathogenic microorganisms, such as *Escherichia coli* ATCC 25922, *Pseudomonas aeruginosa* ATCC 15442, *Staphylococcus aureus* ATCC 25923, and *Candida albicans* ATCC 10231, were used for this test. Tryptic Soy Agar (TSA) (Scharlab) plates were inoculated with a standard inoculum of the target pathogen (0.5 McFarland). After drying the inoculated Petri plate, the biosurfactant and pigment extracts, re-suspended in a 1:4 acetone–water ratio, were spotted (10 μL) onto the TSA. The absence of acetone toxicity was confirmed before testing the antimicrobial activity of the extracts. A gentamicin antibiotic disc (10 µg, Oxoid, Thermo Fisher Scientific, Waltham, MA, USA) and fluconazole (25 µg, Oxoid, Thermo Fisher Scientific, Waltham, MA, USA) served as positive controls. The Petri plates were incubated at 37 °C for 24 h.

## 3. Results and Discussions

Romania is recognized for its abundance of salt deposits, mostly Miocene in origin, which can be found at the surface or shallow depths in different areas (e.g., Transylvanian Depression, Maramureș Depression, Getic Subcarpathians, Moldavian Plateau, Subcarpathian Bend area). These deposits originated through the evaporation of marine water and were subsequently influenced by tectonic activity, which facilitated the upward migration of salt, leading to the formation of salt outcrops, salt banks, saline springs, or hypersaline lakes. These saline resources, some of which continue to be exploited in traditional ways, have been utilized for industrial applications, balneotherapeutic purposes, and human consumption. Over time, people found ways to make use of these resources by building salt wells. Due to their elevated salinity and long-term stability, salt wells represent an ideal environment for the isolation of new moderate and extreme halophilic microorganisms.

### 3.1. Physicochemical Characteristics of Samples

**Figure 1:**
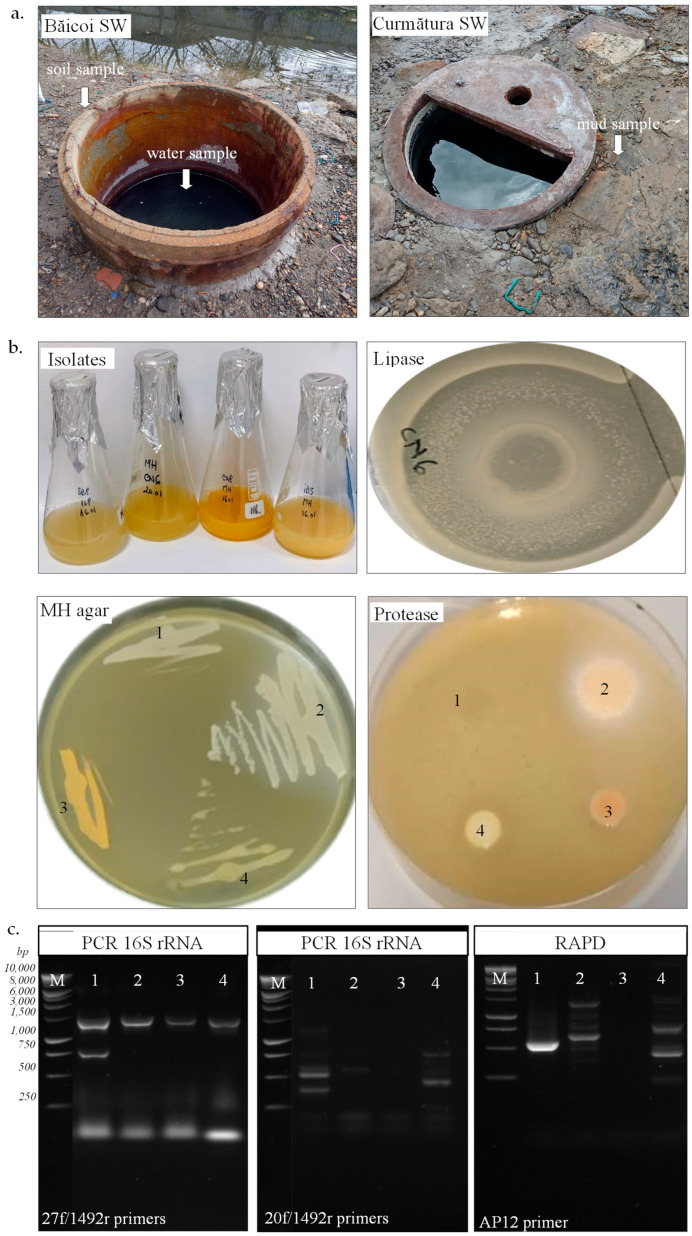
Isolation of halotolerant bacteria from hypersaline samples and their characterization. (**a**). Soil and water samples from Băicoi and mud sample from Curmătura salt well (SW). (**b**). Strain SB8 (1), CN6 (2), CN8 (3), and IB5 (4) grown onto JCM168 and MH medium. (**c**). PCR of 16S rRNA gene using gDNA from SB8 (1), CN6 (2), CN8 (3), and IB5 (4) with bacterial (27f/1492r) or archaeal (20f/1492r) primers; RAPD using gDNA from SB8 (1), CN6 (2), CN8 (3), and IB5 (4) with AP12 primer; 1 kb DNA ladder (M).

The physicochemical analysis of water samples from the brine wells in Băicoi and Curmătura showed differences in salinity, pH, temperature, and chloride ion concentration, likely influenced by local geological conditions and seasonal evaporation effects. The Băicoi salt well, approximately 3 m deep, had a pH of 7.01 and a salinity of 280 g/L, making it a highly concentrated brine. The density was 1.19 g/cm^3^, and the water temperature was 16 °C. The chloride ion concentration measured 191 g/L, reflecting the strong saline nature of the water. The Curmătura salt well, about 2 m deep, had a pH of 7.13 and a slightly higher salinity of 290 g/L. The density was measured at 1.25 g/cm^3^, with a temperature of 19 °C, and a chloride ion concentration of 194 g/L, suggesting a brine composition similar to that of Băicoi, but slightly more concentrated. The high salt content of these two wells justifies their traditional use in food preservation, particularly for making pickles and cheeses. As built wells lined with uncovered concrete, they are exposed to seasonal changes in salinity and potential external contamination (Figure 1a). According to a 2006 ethnographic study on saltwater wells, which classified them based on their level of development and the methods used to capture saline water, the two wells in this study are considered partially developed [42]. The chloride ion content in the soil and mud samples was 42.6 g/L and 56.8 g/L, respectively.

### 3.2. Isolation, Characterization, and Identification of Halophiles

The microbial abundance exhibited a significant difference between the water and sediment samples, with notably higher colony-forming units (CFUs) observed in sediments from both locations. In the Băicoi water sample, bacterial growth was detected at 3 CFU/mL on the MH medium, while no colonies were present on the JCM 168 medium. In Băicoi soil, the microbial counts were considerably higher, with 5.3 × 10^5^ CFU/mg on MH medium and 1.1 × 10^4^ CFU/mg on JCM 168 medium. Curmătura mud samples exhibited 1.2 × 10^5^ CFU/mg on MH medium and 1.2 × 10^4^ CFU/mg on JCM 168 medium, while the water sample showed no microbial growth on these two media. No fungal growth was observed on MH or JCM 168 medium. Out of 30 isolates obtained in pure culture, 4 colonies with a distinct morphology were selected for further characterization. As observed in Table 1, the strains SB8 and IB5 were isolated from soil and water, respectively, from a salt well in Băicoi, while CN6 and CN8 were isolated from mud in Curmătura. Our halophilic isolates exhibited distinct morphological and biochemical traits, highlighting variations in their structural and metabolic characteristics. Growth on selective media further distinguished the strains. SB8 grew on JCM168 agar with 3.4 M NaCl and sodium deoxycholate (0.004% *w*/*v*), confirming its bacterial nature and resistance to bile salts, a trait that differentiates them from extremely halophilic archaea. In contrast, CN6, CN8, and IB5 showed positive growth on MH agar with 1.7 M NaCl and sodium deoxycholate (0.004% *w*/*v*), also suggesting their bacterial characteristics. None of the strains grew on the agar media with chloramphenicol (0.002% *w*/*v*). Colony pigmentation also varied among the isolates, ranging from creamy (SB8) and beige (CN6, IB5) to orange (CN8) (Figure 1b). The optimal growth temperature for most isolates was 30 °C, except for SB8, which grew at 37 °C. Although the SB8 strain was isolated on JCM168 medium at 37 °C, it also grew very well on MH medium at 30 °C. For this reason, all subsequent experiments were conducted on MH medium at 30 °C. Gram staining revealed that strains SB8 and IB5 were Gram-negative, while CN6 and CN8 were Gram-positive. The isolates exhibited distinct cellular morphologies: CN6 displayed a bacillary form and SB8 exhibited a coccoid shape, while CN8 and IB5 demonstrated a coccobacillary morphology. Additionally, motility was observed in CN8 and IB5, whereas SB8 and CN6 were non-motile. Although some differences were observed in their characteristics (e.g., colony color, Gram staining, morphology), all isolates exhibited aerobic and facultative anaerobic growth. The strains SB8 and IB5 were catalase-negative, whereas CN6 and CN8 were catalase-positive. No differences were observed in oxidase activity, as all isolates were oxidase-positive.

The genetic analysis of the four isolates was performed using PCR-based molecular methods, specifically 16S rRNA gene amplification and RAPD fingerprinting (Figure 1c). Successful PCR amplification of the bacterial 16S rRNA gene (1465 bp) using the 27f/1492r primers in all four isolates confirmed their bacterial origin. In contrast, no amplification was observed with archaeal-specific primers (20f/1492r), indicating that none of the isolates belong to the Archaea domain. RAPD fingerprinting of the isolates was performed using the AP12 and AP5 primers. Distinct RAPD profiles (fragments with sizes between 200 and 1500 bp) were observed for strains SB8, CN6, and IB5, whereas CN8 showed no amplification with these primers. Based on the nucleotide sequences of the 16S rRNA gene, strains SB8 and IB5 belong to the *Halomonas* genus (*H. elongata*, *H. stenophila*), strain CN6 belongs to the *Bacillus* genus (*B. altitudinis*), and CN8 belongs to the *Planococcus* genus (*P. rifietoensis*). The results obtained regarding the phylogenetic affiliation of isolated halophilic bacteria are consistent with existing literature data. Previous studies indicate that the *Halomonas* genus is well represented by species such as *Halomonas elongata* and *Halomonas stenophila*, both known for their adaptation to extreme saline environments [7,43,44]. *Halomonas* is a genus of Gram-negative, non-spore-forming bacteria, primarily isolated from marine environments, including deep-sea sediments and hydrothermal vents. In these ecosystems, *Halomonas* can represent up to 10% of the total microbial community [45], underscoring its ecological importance and its ability to adapt to extreme conditions. Regarding the *Bacillus* genus, strain CN6’s identification as *Bacillus altitudinis* aligns with studies describing this species as having a broad ecological distribution [46]. For the *Planococcus* genus, strain CN8, identified as *Planococcus rifietoensis*, shows similarities with previously described species found in saline habitats [46]. The comparison of 16S rRNA sequences with public databases and the observed sequence similarities ranging from 85% to 96% indicate a close relationship but not always complete identity, which is commonly encountered in taxonomic studies based on molecular phylogeny [47].

**Figure 2:**
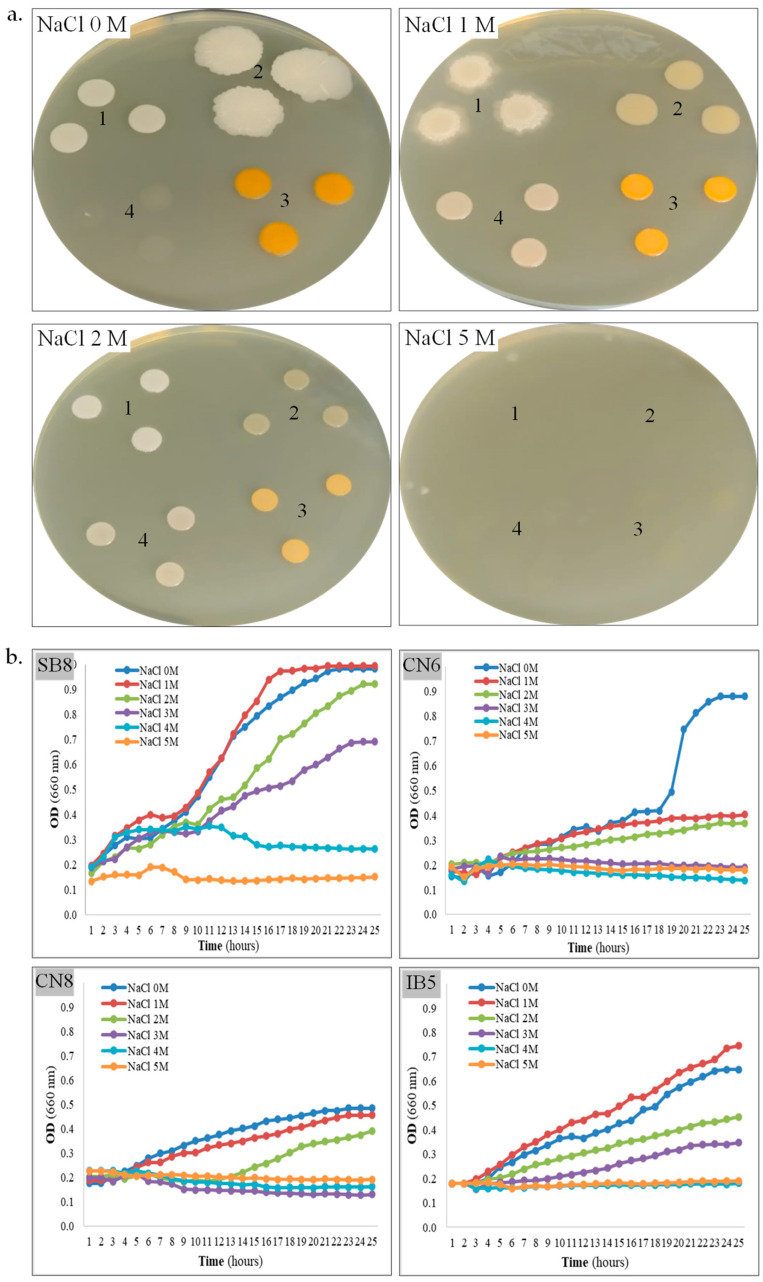
Tolerance of halotolerant bacteria to salt. (**a**). Strain SB8 (1), CN6 (2), CN8 (3), and IB5 (4) grown onto MH agar with 0–5 M NaCl. (**b**). Growth kinetics of strain SB8, CN6, CN8, and IB5 on MH broth with 0–5 M NaCl.

**Halophily and halotolerance.** An important parameter for the laboratory study of newly isolated strains from saline environments is the assessment of salt tolerance on solid and liquid media. The ability of the four bacterial strains to grow on MH agar and MH broth supplemented with NaCl concentrations ranging from 0 to 5 M was further evaluated (Table 1, Figure 2a,b). All isolates revealed growth on MH agar without additional NaCl supplementation (0 M NaCl condition), confirming their halotolerant nature. On MH agar, strain *H. elongata* SB8 exhibited robust growth within the 0–3 M NaCl range, after 48 h. After five days of incubation, colony formation was also observed for this strain at 4 M NaCl, although its growth was slower than at lower concentrations. *B. altitudinis* CN6 and *P. rifietoensis* CN8 demonstrated growth within the 0–2 M NaCl range, with an optimum at 0–1 M NaCl. Strain *H. stenophila* IB5 displayed optimal growth at 1–2 M NaCl and showed limited tolerance at 3 M. Notably, no growth was observed for this bacterium at 0 M NaCl within the first 48 h; however, after five days, colonies emerged on the medium. This delayed response could be attributed to the slower diffusion of nutrients and oxygen in solid media, compared to liquid media, where growth at 0 M NaCl was observed. Additionally, osmotic stress may have prolonged the lag phase, delaying the onset of growth. On a solid medium, colony formation requires active metabolism and rapid cell division, which may further explain this delayed adaptation. None of the bacterial strains exhibited growth on MH agar with 5 M NaCl. Over time, particularly after five days, all colonies displayed distinct morphological variations depending on the salt concentration (Figure 2a). Specifically, pigmentation decreased as NaCl concentration increased, suggesting a metabolic adaptation strategy to osmotic stress. At higher salt concentrations, the colonies became more compact and smaller, indicating slower growth and potential modifications in cell wall composition to regulate water retention. Based on the obtained results, the four newly isolated bacterial strains were classified as halophilic or halotolerant microorganisms. To gain a more dynamic perspective on their adaptation to salinity, bacterial growth kinetics were evaluated in MH broth containing 0–5 M NaCl (Figure 2b). Among the four strains, *H. elongata* SB8 demonstrated the highest salt tolerance. In the 0–3 M NaCl range, it exhibited active growth, reaching high optical density values (OD_660_ 0.92–0.99) within the first 20–24 h. Faster growth was observed at 0 M and 1 M NaCl, as compared with growth in the presence of 2 M or 3 M NaCl. At 4 M NaCl, a delay in the logarithmic growth phase and a reduction in the growth rate were noted, suggesting that the bacterium was affected by osmotic stress and likely required a longer adaptation period. The upper tolerance limit for NaCl was 5 M, with no proliferation of cells. Our results suggest that this bacterium employs efficient osmotic adaptation mechanisms, probably synthesis of compatible solutes and alterations in cell wall structure, which are characteristic of *Halomonas* species [48]. These mechanisms are supported by previous studies on *Halomonas* spp., where the accumulation of osmoprotectants such as ectoine and glycine betaine plays a key role in maintaining intracellular osmotic balance under hypersaline conditions [49]. Another important aspect is that halophilic bacteria have been shown to modify their membrane lipid composition by increasing the proportion of saturated fatty acids and negatively charged phospholipids, which enhance membrane stability and reduce ion leakage under salt stress [22,50]. These changes not only support cell viability at high NaCl concentrations but also contribute to maintaining enzyme functionality and metabolic activity in extreme environments. For the *B. altitudinis* CN6 strain, optimal growth was recorded at 0 M NaCl, reaching maximum optical density values (OD_660_ 0.87) within the first 22–24 h, while over 1 M NaCl, growth was significantly reduced. For *P. rifietoensis* CN8, optimal growth was recorded at 0–1 M NaCl, reaching maximum optical density values (0.45–0.48) within the first 22–24 h; at 1 M and 2 M NaCl, growth was slightly reduced, and the growth curves exhibited a prolonged lag phase and a slower growth rate, especially in 2 M NaCl. Over 3 M NaCl, no growth was detected for *B. altitudinis* CN6 and *P. rifietoensis* CN8. These two strains can be considered moderately tolerant of salts, and our observations are in line with those reported in the literature, where *Bacillus* and *Planococcus* species are described as halotolerant but not strictly halophilic [7,51]. These bacteria do not appear to possess efficient strategies for coping with high osmolarity, which could explain the growth inhibition over 3 M NaCl. These species appear to rely mainly on primary responses, such as potassium ion accumulation and proline (an osmoprotective amino acid) uptake, which may provide short-term protection under conditions of moderate salt stress. Moreover, their membrane structure may be less flexible under saline conditions, reducing the ability to regulate permeability and stabilize cellular functions [19,50]. *H. stenophila* IB5 exhibited active growth in 0–3 M NaCl, with an optimum at 0–1 M NaCl; the highest optical density (0.64–0.74) was obtained in the first 23–24 h. Unlike *B. altitudinis* CN6 and *P. rifietoensis* CN8, *H. stenophila* IB5 showed detectable metabolic activity up to 3 M NaCl, indicating a better capacity for osmotic stress regulation, but still lower than *H. elongata* SB8. These differences in salt tolerance may be explained by the varying ability to accumulate compatible solutes. As with the other strains, growth was completely inhibited over 4 M NaCl. After 24 h of incubation, cell viability was assessed using TTC (Table 1), a metabolic activity indicator of the presence of viable cells, even in the absence of cell division. The results showed that *H. elongata* SB8 and *H. stenophila* IB5 remained viable up to 5 M NaCl, despite the absence of detectable cell growth in the 24 h kinetics assay. *B. altitudinis* CN6 remained viable only up to 2 M NaCl, while *P. rifietoensis* CN8 cells maintained viability up to 3 M NaCl. This difference between growth and viability appears to be a well-documented phenomenon in halotolerant bacteria, where cells can reduce their rate of metabolism under extreme osmotic stress without immediate cell death [7,52,53,54,55].

**Figure 3:**
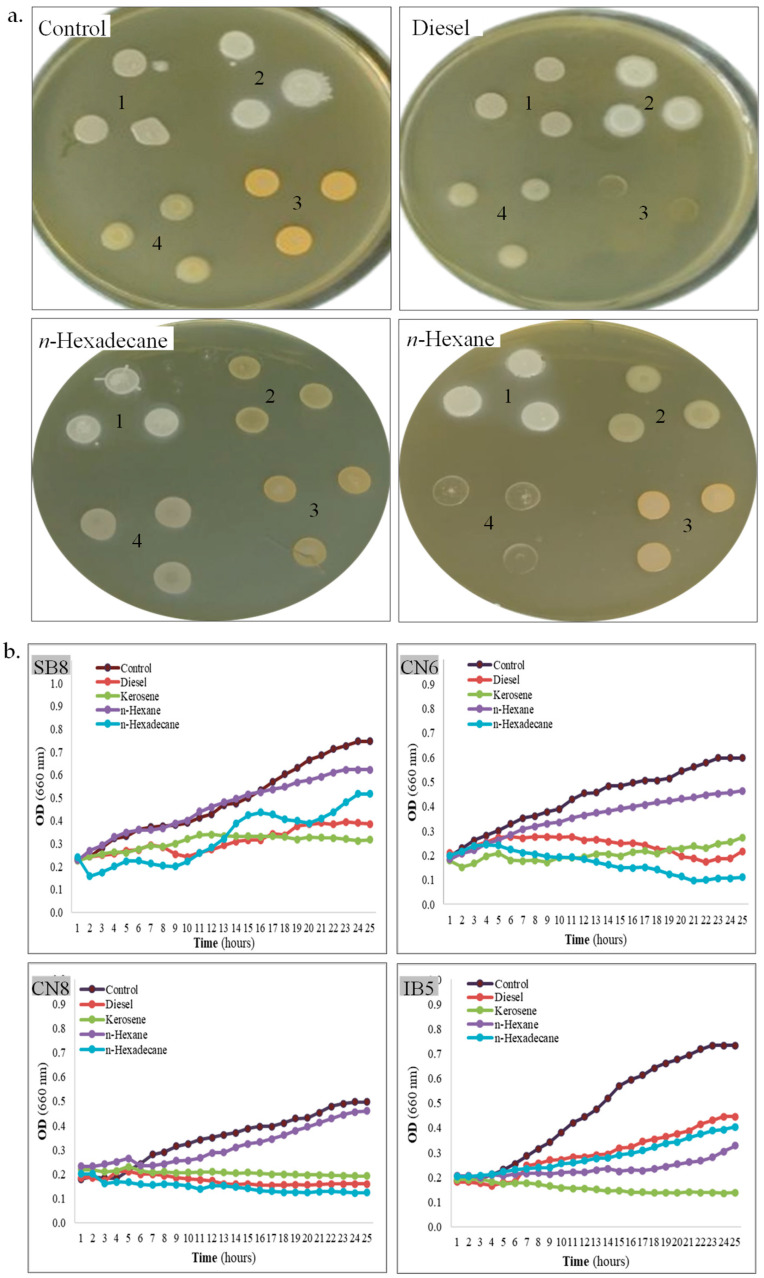
Tolerance of halophiles to hydrocarbons. (**a**). Strain SB8 (1), CN6 (2), CN8 (3), and IB5 (4) grown onto MH agar overlayed or not with hydrocarbons. (**b**). Growth kinetics of strain SB8, CN6, CN8, and IB5 on MH broth with 10% hydrocarbons added or not.

**Hydrocarbon tolerance**. Bacteria isolated from saline environments sometimes exhibit cross-resistance to salinity and hydrocarbons [56], enabling them to survive and thrive under extreme conditions [43]. This adaptation is particularly significant in saline ecosystems, where native microorganisms develop specialized mechanisms to withstand high salt concentrations, and sometimes, they also exhibit high resistance to hydrocarbons [43,57]. The tolerance of the four isolated strains to diesel, kerosene, *n*-hexane, and *n*-hexadecane was assessed on MH agar using the hydrocarbon overlay assay, as well as in MH broth by monitoring the growth kinetics in the presence of the same hydrocarbons, as compared with a control (without hydrocarbons). In the hydrocarbon overlay agar assay (Table 2, Figure 3a), the tolerance of bacterial cells to hydrocarbons varies among different strains. As observed, *H. elongata* SB8 exhibits high tolerance to all tested hydrocarbons, as indicated by confluent growth. Strain *B. altitudinis* CN6 demonstrates good tolerance to diesel, *n*-hexane, and *n*-hexadecane, whereas *P. rifietoensis* CN8 tolerates only *n*-hexane and *n*-hexadecane. *H. stenophila* IB5 shows tolerance to diesel and *n*-hexadecane. Despite belonging to the same genus, *Halomonas*, strains SB8 and IB5 exhibit distinct responses to the tested hydrocarbons. Our results align with previous studies reporting that bacterial tolerance to hydrocarbons is strain-dependent and influenced by hydrocarbon type, cell surface properties, and metabolic capabilities [58,59].

When bacterial growth kinetics were assessed in MH broth supplemented with 10% diesel, kerosene, *n*-hexane, and *n*-hexadecane, each strain exhibited distinct responses to the presence of hydrocarbons (Table 2, Figure 3b). Consistent with the results obtained in the overlay assay, strain *H. elongata* SB8 demonstrated robust growth in the presence of all tested hydrocarbons. Strains *B. altitudinis* CN6 and *H. stenophila* IB5 exhibited substantial growth in the presence of diesel and *n*-hexane, whereas *P. rifietoensis* CN8 showed growth only in the presence of *n*-hexane within the first 24 h. Growth dynamics revealed a gradual adaptation to hydrocarbons, characterized by an initial lag phase followed by continuous growth after 6–8 h. Previous studies on various bacteria isolated from sites contaminated with petroleum and its derivatives suggest that an adaptation period is required for the activation of specific enzymes involved in hydrocarbon degradation [35,38]. However, in the growth kinetics assay, strain-specific differences were observed. In the viability assay conducted after 24 h of hydrocarbon exposure, all the tested bacteria remained viable, except strain *H. stenophila* IB5. For this bacterium, no growth was observed in the presence of kerosene and no viable cells were identified. These findings suggest that while some bacteria can survive in the presence of certain hydrocarbons, they may not be capable of proliferating on them without an extended lag phase. Notably, strain *P. rifietoensis* CN8 demonstrated growth in diesel-containing broth despite its inability to grow on diesel agar, indicating that the broth medium may mitigate diesel toxicity or enhance substrate availability. Strain *H. stenophila* IB5 was completely inhibited by kerosene, highlighting its high sensitivity to this compound. *H. elongata* SB8 and *B. altitudinis* CN6 exhibited the highest growth rates, as reflected by increased OD_660_ values, demonstrating their strong adaptive capacity in hydrocarbon-contaminated environments. A comparison of bacterial behavior in MH agar and MH broth revealed notable differences. In agar medium, direct contact with hydrocarbons may exacerbate their toxic effects and inhibit bacterial growth. Conversely, in the MH broth, aeration facilitates hydrocarbon dispersion, reducing toxicity and enhancing bioavailability, thereby supporting bacterial survival and metabolism. These results indicate that the biodegradation process is more effective under dynamic conditions than under static ones. Moreover, bacterial tolerance to hydrocarbons appears to be determined by intrinsic physiological traits, as well as by the chemical properties and environmental hydrocarbon availability. The observed variability in tolerance to toxic hydrocarbons may be attributed to differences in cell membrane composition and the ability to produce secondary metabolites, as reported in other bacteria belonging to the genera *Pseudomonas*, *Achromobacter*, *Acinetobacter*, *Bacillus*, and *Stenotrophomonas* [35,36,37]. In this context, exploring the biotechnological potential of new halophilic or halotolerant bacteria in bioremediation and other industrial applications is necessary for better understanding of their resistance and adaptation mechanisms [60].

### 3.3. Biomolecule Production by Halophiles

**Extracellular hydrolytic enzymes.** Bacteria isolated from saline environments are known to produce extracellular hydrolytic enzymes, which are essential for breaking down complex substrates and adapting to osmotic stress [14,39,61,62]. Consequently, we evaluated the capacity of newly halotolerant isolated bacterial strains to produce several extracellular hydrolytic enzymes involved in the degradation of various organic substrates (Table 3). The enzymatic activities tested included lipase (Tween-80 hydrolysis), protease (casein hydrolysis), and polysaccharide-degrading enzymes, such as amylase (starch hydrolysis), cellulase (CMC hydrolysis), xylanase (xylan hydrolysis), and pectinase (pectin hydrolysis). The results revealed that each strain tested had unique enzymatic profile, highlighting differences in their metabolic capacity and substrate utilization, potentially influenced by their isolation sources. Combined hydrolytic activities were observed in all the bacterial isolates. Although both *H. elongata* SB8 and IB5 belong to the genus *Halomonas*, they showed differences in their ability to produce hydrolytic enzymes. *H. elongata* SB8 displayed lipolytic and amylolytic activity, whereas *H. stenophila* IB5 exhibited a broad enzymatic profile, producing proteases, cellulases, and xylanases. The capacity of *Halomonas* species to produce lipases is well documented, particularly in strains from hypersaline and hydrocarbon-contaminated environments, in which lipases play a key function in the biodegradation of hydrophobic compounds [63,64]. The strain CN6, identified as *B. altitudinis*, has the highest capacity to degrade multiple substrates. Thus, it exhibited strong lipolytic activity, with a hydrolysis zone exceeding 15 mm in diameter (Figure 1b). Additionally, *B. altitudinis* CN6 was found to produce protease, cellulase, and xylanase, indicating a diverse metabolic capacity for degrading complex organic matter, a characteristic that is typically found in halotolerant *Bacillus* species [62]. The strain CN8, identified as *P. rifietoensis*, exhibited protease and cellulase activity, but with a more limited metabolic range compared to *B. altitudinis* CN6. Planococcus species are well known for their ability to survive in extreme environments and their capacity to degrade polysaccharides, but only some isolates have been documented to exhibit proteolytic activity [62]. Notably, none of the tested halotolerant strains presented pectinolytic activity. This is consistent with data from the literature, which shows that pectinase production is generally low among halophilic and halotolerant bacteria, as these microorganisms predominantly produce proteases, lipases, and cellulases rather than enzymes involved in pectin degradation [65,66].

**Figure 4:**
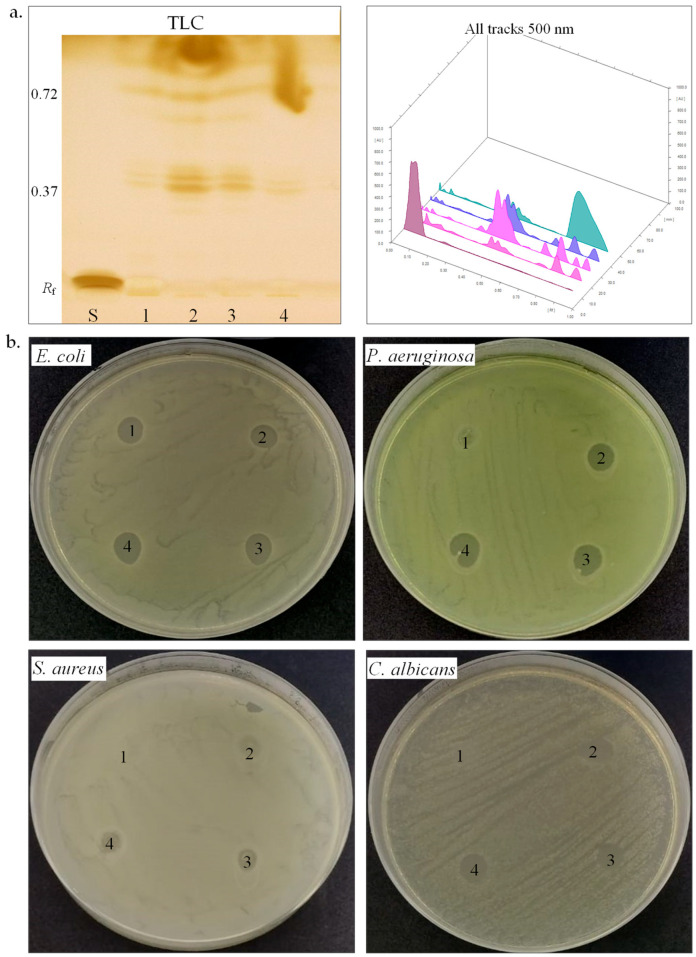
Biosurfactant production by halotolerant bacteria. (**a**). TLC of biosurfactant extracted from strain SB8 (1), CN6 (2), CN8 (3), and IB5 (4) grown on MH broth, retardation factor (*R*_f_), standards (S, L-Rhamnose), TLC plate observed and scanned under visible light. (**b**). Antimicrobial activity of biosurfactants extracted from strain SB8 (1), CN6 (2), CN8 (3), and IB5 (4) on pathogenic microorganisms.

**Biosurfactants.** Biosurfactants play a significant role in microbial adaptation to harsh environments, especially among halotolerant and halophilic bacteria, by facilitating membrane stability, substrate solubilization, and hydrocarbon emulsification [67]. We further studied the biosurfactant-producing potential of the four halotolerant bacterial strains (Table 3, Figure 4a,b). The emulsification index (*E*_24_) serves as an indirect screening method for biosurfactant production [35,36,37]. *H. elongata* SB8 and *B. altitudinis* CN6 demonstrated maximum emulsification activity (*E*_24_ 100%), whereas *P. rifietoensis* CN8 and *H. stenophila* IB5 exhibited no detectable emulsification. The two newly isolated *Halomonas* strains displayed a higher emulsification index than those reported for other *Halomonas* strains. *Halomonas desertis* G11 produced a biosurfactant with an *E*_24_ of 69.4% [68], lower than *H. elongata* SB8, suggesting SB8 secretes a distinct, possibly more efficient glycolipid or a glycolipid–exopolysaccharide complex. Similarly, *Halomonas* sp. BS4 was identified as an excellent glycolipid biosurfactant producer [69]. The unique phytanylglycerol-based membrane lipids of *Halomonas* species could enhance their surface-active properties [70]. *Bacillus* biosurfactants are primarily lipopeptides like surfactin, fengycin, and iturin, which effectively reduce surface tension and enhance interaction with hydrophobic substrates, including petroleum hydrocarbons. While *Bacillus* is an efficient biosurfactant producer, its emulsification capacity depends on substrate type and biosurfactant structure [71,72]. In contrast, *P. rifietoensis* CN8 did not produce biosurfactants detectable by the emulsification method, which is consistent with studies in the literature reporting that this genus is not a typical biosurfactant producer [73]. To confirm biosurfactant production, the methylene blue method was utilized to detect anionic biosurfactants. The absorption at 625 nm was highest for *H. elongata* SB8 (1.94), followed by *H. stenophila* IB5 (1.76) and *B. altitudinis* CN6 (1.50), while *P. rifietoensis* CN8 showed a lower level (1.42). These values indicate that all four strains produced surfactant compounds, although their efficiency as emulsifying agents varied. Notably, while *H. stenophila* IB5 displayed a strong signal in this test, it did not stabilize the emulsion, suggesting either a low biosurfactant concentration or an ineffective chemical structure for emulsification. While the methylene blue and emulsification assays confirmed biosurfactant activity, the CTAB agar assay showed no evidence of rhamnolipid production in any of the tested strains. To gain further insight into the chemical composition of the biosurfactants produced by the strains from the genera *Halomonas*, *Bacillus*, and *Planococcus*, HPTLC analysis was conducted. After TLC plate scanning and visualization under UV light before derivatization, up to four spots with retention factors (*R*_f_) ranging from 0.37 to 0.72 were observed. Subsequently, scanning the plate after derivatization with iodine vapors and orcinol solution allowed for the visualization of colored spots, indicating the presence of different biosurfactant components. The positive reaction to iodine vapors (yellow-brown spots) confirmed the presence of lipid fractions, while the positive reaction to orcinol solution (brown-red spots) confirmed the presence of a carbohydrate component in the structure of the biosurfactants. In our analysis, the TLC profiles revealed the following *R*_f_ values: for *H. elongata* SB8, 0.39, 0.42, and 0.72; for *B. altitudinis* CN6, 0.38, 0.41, 0.62, and 0.71; for *P. rifietoensis* CN8, 0.38, 0.42, 0.67, and 0.72; and for *H. stenophila* IB5, 0.37, 0.40, and 0.72. The *R*_f_ value of 0.72, detected in all strains, corresponds to glycolipid fractions, which are commonly produced by halotolerant and halophilic bacteria [74]. The *R*_f_ values between 0.38 and 0.42 indicate lipid-based biosurfactant fractions, which were identified as phospholipids or lipopeptides [75,76]. Interestingly, in our analysis, the strains *P. rifietoensis* CN8 and *H. stenophila* IB5 exhibited *R*_f_ values ranging between 0.37 and 0.72 but did not show clear emulsification activity. This indicates that either the concentration of biosurfactants produced was insufficient, or the molecular structure of the compounds may not be efficient enough to stabilize emulsions. Biosurfactants produced by some bacteria could have antimicrobial activity [72,77]. Thus, we further tested by the agar diffusion method against pathogenic microorganisms such as *E. coli* ATCC 25922, *P. aeruginosa* ATCC 15442, *S. aureus* ATCC 25923, and *C. albicans* ATCC 10231 (Table 3, Figure 4b). Gentamycin (10 µg) was used as a positive control for antibacterial activity, while fluconazole (25 µg) served as the positive control for antifungal activity. Acetone was used as the negative control and showed no inhibition zones against pathogenic microorganisms. All the biosurfactant extracts exhibited an inhibitory effect against *E. coli* and showed no effects against *S. aureus*. The biosurfactant extract from *H. elongata* SB8 had no effect against *P. aeruginosa*, while the biosurfactants extracted from *B. altitudinis* CN6, *P. rifietoensis* CN8, and *H. stenophila* IB5 showed an inhibitory effect against this bacterial pathogenic strain. In the case of *C. albicans*, the extracts from *H. elongata* SB8 and *B. altitudinis* CN6 had no antifungal activity, whereas the extracts from *P. rifietoensis* CN8 and *H. stenophila* IB5 demonstrated positive results against this fungus. Overall, the results showed that biosurfactants produced by our halotolerant bacteria have different properties, varying from strain to strain, which is reflected in their different emulsification capacities and antimicrobial activity.

**Figure 5:**
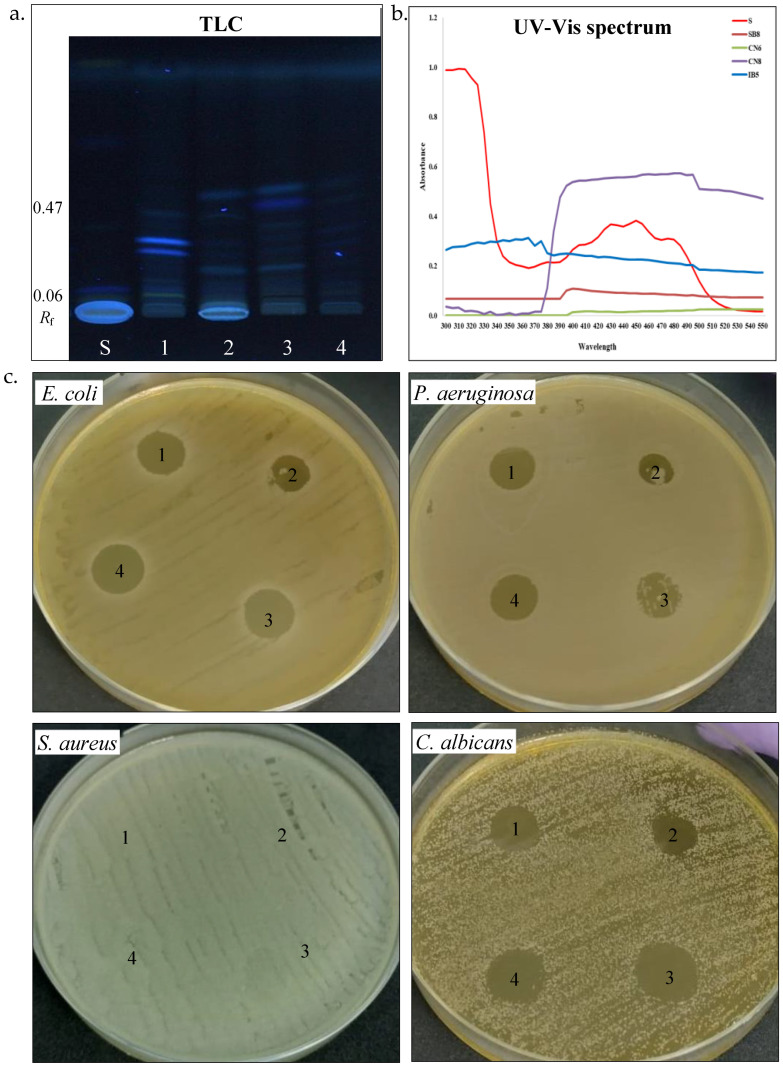
Carotenoid pigments’ production by halotolerant bacteria. (**a**). TLC of carotenoids extracted from strain SB8 (1), CN6 (2), CN8 (3), and IB5 (4) grown on MH broth; retardation factor (*R*_f_), carotenoid standards (S), TLC plate observed under UV light. (**b**). UV-Vis spectrum of carotenoids (300–550 nm). (**c**). Antimicrobial activity of carotenoids extracted from strain SB8 (1), CN6 (2), CN8 (3), and IB5 (4) on pathogenic microorganisms.

**Carotenoid pigments.** Carotenoids are common pigments found in salt-loving bacteria, where they help protect cells from oxidative stress and harmful UV radiation [49]. UV-Vis spectrophotometry and thin-layer chromatography (TLC) were used to study the carotenoid pigment production by the four halotolerant bacterial strains (Table 3, Figure 5a–c). UV–Vis spectroscopy analysis of pigments extracts from *P. rifietoensis CN8* revealed absorption peaks within the 395–500 nm range, with prominent maxima at 480–485 nm, confirming the presence of carotenoids. This finding aligns with Kushwaha et al. [21], who reported that *Planococcus maritimus* produced carotenoids with absorption maxima at 437 nm and 458 nm, corresponding to β-carotene and other structurally related carotenoids. For *H. stenophila* IB5, weak absorption was detected around 370 nm, suggesting the presence of a distinct pigment, possibly a precursor carotenoid, such as phytofluene, which absorbs in the 350–380 nm range. Interestingly, TLC revealed visible pigment bands for all bacterial strains, including *B. altitudinis* CN6 and *H. elongata* SB8, despite the absence of detectable absorption peaks in their UV-Vis spectra. This discrepancy could be attributed to differences in the sensitivity of the methods, as TLC can separate even trace amounts of pigments that may be below the detection limit of UV-Vis spectroscopy. A similar observation was reported by Fariq et al. (2019) [49] for *Halomonas aquamarina*, where carotenoids were detected via TLC but were present in low concentrations in solvent extracts analyzed by UV-Vis spectrophotometry. To further quantify carotenoid production, total carotenoid concentrations were determined for the four strains. *P. rifietoensis* CN8 exhibited the highest concentration (453.44 µg/mL), followed by *H. stenophila* IB5 (162.8 µg/mL). In contrast, *H. elongata* SB8 produced a lower amount (64.96 µg/mL), while *B. altitudinis* CN6 had the lowest concentration (15.76 µg/mL). Despite the lack of UV-Vis absorption in some strains, TLC confirmed the presence of carotenoid pigments in all four strains, with distinct *R*_f_ values indicating variations in pigment composition. The cream-colored pigments of *H. elongata* SB8 exhibited the narrowest *R*_f_ range (0.06–0.35), suggesting a predominance of more polar carotenoids or biosynthetic intermediates, such as phytoene or phytofluene. In contrast, the beige pigment extract of *B. altitudinis* CN6 displayed bands across a broader range (*R*_f_ 0.07–0.43), indicative of a more diverse pigment composition, potentially including zeaxanthin-like compounds. The orange pigment extract of *P. rifietoensis* CN8 and the cream-colored pigments of *H. stenophila* IB5 exhibited the highest *R*_f_ values (up to 0.46 and 0.47, respectively), suggesting the presence of more hydrophobic carotenoids, potentially similar to β-carotene or its derivatives. The pigment extracts were further evaluated for antimicrobial activity using the agar diffusion method against the same four pathogenic microorganisms (Table 3, Figure 5c). The results demonstrated that all pigment extracts exhibited antifungal activity against *C. albicans*. Notably, *H. elongata* SB8 and *H. stenophila* IB5 demonstrated an inhibitory effect against *E. coli* and *P. aeruginosa*. In contrast, the *B. altitudinis* CN6 extract did not inhibit any bacterial strain, while the *P. rifietoensis* CN8 pigment inhibited only *E. coli*. None of the pigment extracts exhibited inhibitory activity against *S. aureus*. These results suggest that the antimicrobial properties of the pigments may be species-specific, with a more pronounced effect against Gram-negative bacteria and fungal pathogens. Fariq et al. [49] reported antimicrobial activity in pigments produced by a *Halomonas* strain, demonstrating inhibitory effects against a broad spectrum of microorganisms, including Gram-negative and Gram-positive bacteria, and fungi. These findings suggest that carotenoid pigments produced by *Halomonas* species may contribute to antimicrobial activity, potentially serving as a defense mechanism against competing microorganisms.

## 4. Conclusions

The findings of this study represent a significant advancement in elucidating the biotechnological potential of halophilic and halotolerant bacteria isolated from two unexplored saline environments. Phenotypic and molecular analyses identified the halotolerant bacterial strains *Halomonas elongata* SB8, *Halomonas stenophila* IB5, *Bacillus altitudinis* CN6, and *Planococcus rifietoensis* CN8, which exhibited the ability to produce hydrolytic enzymes, biosurfactants, and carotenoids. These metabolic traits highlight their potential for industrial, pharmaceutical, and environmental biotechnology applications. Among the four isolated bacteria, *B. altitudinis* CN6 exhibited broad enzymatic activity, producing lipase, protease, cellulase, and xylanase, suggesting its suitability for biocatalysis, particularly under high-salinity conditions, and for biomass degradation. Notably, *H. elongata* SB8 displayed the highest salt tolerance and hydrocarbon resistance, positioning it as a strong candidate for bioremediation. Additionally, both *H. elongata* SB8 and *B. altitudinis* CN6 were found to synthesize biosurfactants with a high emulsification index, further highlighting their biotechnological relevance. Regarding pigment biosynthesis, *P. rifietoensis* CN8 produced a significant concentration of carotenoids, exhibiting antifungal activity against *Candida albicans* and antibacterial efficacy against *Escherichia coli*. Furthermore, all biosurfactant and pigment extracts demonstrated antibacterial properties against Gram-negative reference strains and the fungal reference strain. The obtained results are particularly relevant to sustainable development, as the halotolerant microorganisms identified in this study offer promising avenues for eco-friendly biotechnological applications. The biosynthesis of valuable biomolecules such as hydrolytic enzymes, biosurfactants, and carotenoids is an essential step in the production of sustainable, bio-based products, which are environmentally friendly alternatives to traditional chemical-based processes. The potential for utilizing these microorganisms in bioremediation efforts, particularly in saline or polluted environments, supports sustainable practices by mitigating the harmful effects of pollutants while promoting ecosystem restoration. However, further research is required to optimize culture conditions to enhance metabolite production and to elucidate the precise mechanisms underlying the biosynthesis of these bioactive compounds.

## Figures and Tables

**Table 1:** Phenotypic and molecular characterization of halotolerant bacteria.

Characteristics	Strain			
	SB8	CN6	CN8	IB5
Isolation source	Băicoi soil	Curmătura mud	Curmătura mud	Băicoi water
Growth on:				
JCM168 agar	+	−	−	−
MH agar	+	+	+	+
Chloramphenicol (0.002%)	−	−	−	−
Sodium deoxycholate (0.004%)	+	+	+	+
Color of colonies	creamy	beige	orange	beige
Temperature growth (°C)	37	30	30	30
Gram	−	+	+	−
Cell shape	cocci	bacilli	coccobacilli	coccobacilli
Motility	−	−	+	+
Respiratory type	A, FA	A, FA	A, FA	A, FA
Catalase	−	+	+	−
Oxidase	+	+	+	+
Salt tolerance capacity (growth on/in MH-NaCl)				
MH-NaCl agar (CF)	0–4 M	0–2 M	0–2 M	1–3 M
MH-NaCl broth (GK)	0–3 M	0–2 M	0–2 M	0–3 M
MH-NaCl broth (CV)	0–5 M	0–2 M	0–3 M	0–5 M
PCR 16S rRNA gene using primers for				
Bacteria (1465 bp)	+	+	+	+
Archaea (1472 bp)	−	−	−	−
RAPD using primer:				
AP12	+	+	−	+
AP5	+	+	−	+
16S rRNA gene sequence identity	*Halomonas elongata*	*Bacillus* *altitudinis*	*Planococcus rifietoensis*	*Halomonas stenophila*

Growth on MH-NaCl agar, colony formation (CF); growth in MH-NaCl broth, growth kinetics (GK) and cell viability (CV); positive reaction (+), negative reaction (−).

**Table 2:** Tolerance to hydrocarbons of halotolerant bacteria.

Characteristics	Strain			
	SB8	CN6	CN8	IB5
Hydrocarbons tolerance (growth on MH agar, CF)				
Control	+	+	+	+
Diesel	+	+	−	+
Kerosene	+	−	−	−
*n*-Hexane	+	+	+	−
*n*-Hexadecane	+	+	+	+
Hydrocarbons tolerance (growth in MH broth, GK, CV)				
Control	+, +	+, +	+, +	+, +
Diesel	+, +	+, +	−, +	+, +
Kerosene	+, +	−, +	−, +	−, −
*n*-Hexane	+, +	+, +	+, +	+, +
*n*-Hexadecane	+, +	−, +	−, +	+, +

Growth on MH agar, colony formation (CF); growth in MH broth, growth kinetics (GK) and cell viability (CV); positive reaction (+), negative reaction (−).

**Table 3:** Biomolecule production by halotolerant bacteria.

Characteristics	Strain			
	SB8	CN6	CN8	IB5
Extracellular hydrolase production:				
Lipase (Tween-80)	+	+	−	−
Protease (casein)	−	+	+	+
Amylase (starch)	+	−	−	−
Cellulase (carboxymethylcellulose)	−	+	+	+
Xylanase (xylan)	−	+	−	+
Pectinase (pectin)	−	−	−	−
Biosurfactants production				
Emulsification index (*E*_24_, %)	100	100	−	−
Methylene blue (OD_625_)	1.94	1.50	1.42	1.76
CTAB	−	−	−	−
HPTLC (*R*_f_)	0.39–0.72	0.38–0.71	0.38–0.72	0.37–0.72
Antimicrobial activity				
*E. coli* ATCC25922	+	+	+	+
*P. aeruginosa* ATCC 15442	−	+	+	+
*S. aureus* ATCC25923	−	−	−	−
*C. albicans* ATCC 10231	−	−	+	+
Carotenoid pigments production				
Total concentration (μg/mL)	64.96	15.76	453.44	162.80
HPTLC (*R*_f_)	0.06–0.35	0.07–0.43	0.08–0.46	0.07–0.47
Antimicrobial activity				
*E. coli* ATCC25922	+	−	+	+
*P. aeruginosa* ATCC 15442	+	−	−	+
*S. aureus* ATCC25923	−	−	−	−
*C. albicans* ATCC 10231	+	+	+	+

Positive reaction (+), negative reaction (−).

## Data Availability

The original contributions presented in this study are included in the article. Further inquiries can be directed to the corresponding authors.
